# Structural Effects of Methionine Oxidation on Isolated Subdomains of Human Fibrin D and αC Regions

**DOI:** 10.1371/journal.pone.0086981

**Published:** 2014-01-27

**Authors:** Patrick R. Burney, Nathan White, Jim Pfaendtner

**Affiliations:** 1 Department of Chemical Engineering, University of Washington, Seattle, Washington, United States of America; 2 Puget Sound Blood Center Research Institute and Division of Emergency Medicine, University of Washington, Seattle, Washington, United States of America; German Research School for Simulation Science, Germany

## Abstract

Oxidation of key methionine residues on fibrin leads to altered fibrin polymerization producing severely altered fibrin gel structure and function. This is important because fibrinogen and its modification by oxidative stress have been implicated as key contributors to both pathological thrombotic and hemorrhagic diseases ranging from cardiovascular thrombosis to the acute coagulopathy of trauma. However, how oxidation leads to altered fibrin polymerization remains poorly understood at the molecular level. We have applied a powerful and novel well-tempered ensemble parallel tempering (PT-WTE) technique along with conventional molecular dynamics (MD) simulation to investigate the molecular-level consequences of selective methionine oxidation of fibrinogen. We offer new insights into molecular mechanisms of oxidation-induced changes in fibrin polymerization, while focusing on the D region knob ‘B’ and hole ‘b’ interaction and αC-domain interactions, both of which are hypothesized to contribute to the lateral aggregation mechanism of fibrin fibrils. Methionine oxidation did not alter the native state or the stability of a bound knob ‘B’ surrogate when interacting with hole ‘b’ in the D region. However, applying PT-WTE simulation to a human homology model of the bovine N-terminal subdomain fragment from the αC-domain revealed that methionine oxidation altered the conformation of the hairpin-linking region to favor open rather than closed hairpin structures. We attribute this alteration to the disruption of the hairpin-linking region's conformation, with oxidation increasing the radius of gyration for this segment. This result is in agreement with experimental data demonstrating decreased fibrin protofibril lateral aggregation when methionine oxidation is present in the same αC-domain fragment. Therefore, single methionine oxidation within the αC-domain is a likely molecular mechanism.

## Introduction

During blood clot formation, the plasma protein fibrinogen is normally converted by thrombin to fibrin monomers that self-polymerize both linearly – forming long fibrils – and laterally, increasing fibrin fiber diameter and forming networks of fibers. These branching fibers make up the 3-D scaffold of blood clots and confer strength, flexibility and structure to clots so they can adhere to wounds and stop bleeding. Increasing fibrinogen concentration is typically associated with a condensed clot structure, thinner fibrin fibers, and increased clot stiffness [1]. This structure-concentration relationship may mediate the long recognized positive association between fibrinogen concentration and the risk of thrombotic cardiovascular disease, including acute myocardial infarction [2] and microvascular disease in diabetes mellitus [3].

There is also evidence that post-translational modification of fibrinogen may contribute to cardiovascular disease. Glycosylation of fibrinogen in diabetics has been hypothesized as a possible mechanism of decreased fibrin gel permeation despite normal fibrinogen concentration [4]. Fibrinogen is also among the most sensitive plasma proteins to post-translational modification by oxidative stress [5]. This susceptibility is important because there is a direct link between fibrinogen oxidation and/or nitration and fibrin polymerization [6], [7]. Depending on the type and extent of modification, these changes can induce a prothrombotic or antithrombotic clot phenotype [8], [9]. Fibrinogen oxidation has been associated with increased cardiovascular events in chronic kidney disease patients [10], [11] and fibrinogen nitration has been associated with myocardial infarction, possibly due to its induction of a prothrombotic fibrin clot phenotype [12]. This evidence highlights the potential for oxidative modification of fibrinogen to contribute to the mechanism of thrombotic cardiovascular diseases. However, little is known of how the molecular events associated with oxidation can alter fibrin polymerization.

Recent evidence uncovered by Weigandt et al. [13], shows that selective oxidation of methionine on fibrinogen – forming methionine sulfoxide – can inhibit lateral aggregation of fibrin protofibrils after activation by thrombin and produces an ultra-thin fibrin fiber network with weakened mechanical properties and increased resistance to fibrinolysis. They found that oxidation of methionine primarily occurs at two positions – residue 476 of the α chain (αMet476) and 367 of the β chain (βMet367). Understanding the mechanism of fibrin lateral aggregation is an area of active research and many studies have indicated these specific sites are important, with αMet476 belonging to the structured portion of the αC region [14] and βMet367 being part of hole ‘b’ of the fibrin D region [15], [16], [17]. Their finding of selective methionine oxidation in association with altered fibrin polymerization and altered gel structure offers an opportunity to examine more closely the effect of oxidation on fibrin polymerization via a novel pathway in addition to the basic mechanism of fibrin polymerization. An important first goal would be to discover the mechanism by which oxidation influences the behavior of fibrin at the molecular level.

Despite the wealth of mesoscale information about the influence of oxidation on fibrin gel structure, little experimental evidence is available at the molecular scale. With the current understanding of the multiple proposed mechanisms of fibrin lateral aggregation along with the crystal and NMR structures of fibrin, molecular simulation provides an avenue to further understanding of these mechanisms and clarifying which portion of fibrin is responsible for the observed gel characteristics. In this study we utilize classical molecular dynamics (MD) in addition to parallel tempering MD (PT) with the recently developed well-tempered ensemble (PT-WTE) to independently examine the effect of βMet367 and αMet476 oxidation on the human fibrin D region and the N-terminal subdomain of the human αC-domain, respectively. Specifically, MD simulation of a fibrin monomer fragment is used to examine the effect oxidation has on the structure of hole ‘b’ and the stability of a bound knob ‘B’ surrogate in the D region. Enhanced sampling PT-WTE simulations are used for the first time without additional metadynamics bias on the protein degrees of freedom in a fully solvated representation of an αC-subdomain in order to explore the conformational ensemble of this protein fragment and characterize the differences created by methionine oxidation.

## Methods

### Molecular Structures

**Figure 1:**
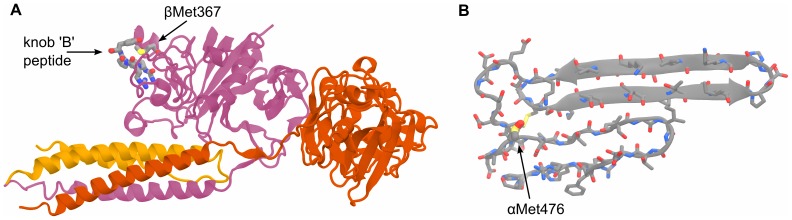
Molecular representations of the initial structures of (A) the D region corresponding to PDB 2Z4E and (B) the αC-subdomain homology model. The methionine of interest in each system is in a location critical to the hypothesized lateral aggregation mechanisms. The D region βMet367 is in direct contact with the bound knob ‘B’ peptide and is part of hole ‘b’. αMet476 is part of the “hairpin-linking region”. The highly helical (coiled-coil) segment of the D region system is part of the fibrin E region.

The D region structure from human fibrin was retrieved from the Protein Data Bank (PDB) entry 2Z4E [18]. The structure contains only a portion of the coiled-coil E region but contains the entirety of the D region (see Figure 1A), including both hole ‘a’ and hole ‘b’ with bound GHRP and GHRPY peptides, respectively. These peptides represent surrogates of knob ‘B’ of fibrin, which has been shown to associate with fibrin holes ‘a’ and ‘b’ [17]. The structure was chosen over the more complete structure, found as PDB entry 3GHG [19], in order to limit system the size to a single D region without having to arbitrarily choose a location to truncate the coiled-coil region. The Cα root mean square deviation (RMSD) between the matching residues of 2Z4E and 3GHG (662 residues) is 0.10 nm, showing that the protein backbone coordinates for these structures are consistent. To avoid confusion, we follow the recommended nomenclature for fibrin and fibrinogen in this paper [20].

In order to examine the effects of methionine oxidation on hole ‘b’ influenced lateral aggregation – that methionine sulfoxide either disrupts the native conformation or the stability of the bound knob ‘B’– a total of four systems were built. These systems include all combinations of the fibrin fragment with or without the knob ‘B’ surrogates and with or without oxidation of βMet367. The first system built was that with methionine not oxidized and with bound knob ‘B’ surrogates. This initial system was then used to create the other three systems, either by addition of the methionine sulfoxide's oxygen, and/or deletion of the knob ‘B’ surrogates. The GHRPY fragment found in hole ‘b’ of the crystal structure was modified to GHRPL by replacing the tyrosine side chain with that of leucine in order to replicate the N-terminal sequence of the human fibrin β chain, knob ‘B’. The protein structure for each system was solvated in Amber's [21] leap with an equilibrated TIP3P [22] water box to a maximum of 2.5 nm from any protein atoms. This large padding allows for some rotation of the protein before an undesirable interaction with its own periodic image. Using leap, chlorine ions were added by replacing water molecules to neutralize the system charge. The initial box dimensions for each system was approximately 8.8×8.8×15.0 nm with approximately 110,000 atoms. All of the D region systems retained the glycosylation and bound calcium ions present in the crystal structure.

The structured portion of the human αC region, termed the αC-domain, has yet to be resolved by X-ray crystallography or NMR experiments. To date, conjecture about the N-terminal subdomain of the αC-domain's lateral aggregation mechanism and estimates of its structure comes from homology models built from recombinant bovine αC-domain fragments, which has been resolved by solution NMR [23]. The homology model used for the initial protein coordinates in this study was obtained from the Medved group and is discussed in their recent publication [24]. The model consists of 55 amino acids (441-496α) and possesses two beta hairpins arranged into a beta-sheet like interaction, a disulfide bonded cysteine pair between one hairpin and the connecting segment, and a single methionine near the connecting segment on the larger hairpin (see Figure 1B). Additionally, two homology models were built using the primary sequence of the human α chain [25] using the homology model program Modeller [26]. Of the 10,000 homology models generated, the best models as determined by Rosetta [27] and DOPE score were tested alongside the Medved model for stability during preliminary 300, 350 and 400 K MD simulations in the NVT ensemble. Of the three models, the Medved model maintained the lowest Cα root mean squared deviation (RMSD) after 25 ns and was subsequently chosen for the simulations described herein.

Two PT-WTE simulations using the homology model of the N-terminal subdomain of the αC-domain were used in this study to examine the effects of oxidation. They correspond to the αC-subdomain with and without αMet476 oxidation. Similar to the D region simulations discussed above, the methionine sulfoxide residue was built by adding the methionine sulfoxide oxygen in a position corresponding to the geometry optimization of methionine sulfoxide in vacuum. Using leap, the initial coordinates of each model were solvated with TIP3P water molecules to a box dimension of 6.0×6.0×10.0 nm and solvent molecules were replaced with chlorine ions to neutralize the systems.

### Molecular Dynamics Simulations

Simulations described in this study were completed in GROMACS 4.5 [28] with the proteins parameterized using the Amber 99SB-ildn force field [29]. The glycosylation and associated asparagine residues were parameterized with the GLYCAM06 [30] force field. Methionine sulfoxide residues, which represent the oxidized state of methionine, were parameterized in Amber's antechamber using the GAFF force field in addition to RESP charges calculated from a Gaussian 09 structure optimization at the HF-6/31-g*(d) level of theory.

The systems were first subjected to 1000 steps of conjugate gradient minimization with all protein positions restrained followed by an additional 4000 steps with no restraints. The minimized system was then gradually heated from 0 to 310 K in increments of 10 K for 2000, 2 fs time steps of NVT simulation at each temperature. The αC-subdomain coordinates were further heated to 470 K, again by increments of 10 K, with the final coordinates at each 10 K increment saved, to generate the initial coordinates for each parallel tempering replica (more details below). For each system the long range Van der Waals forces were smoothly reduced to zero, between the range of 0.9 to 1.0 nm, by shifting the Lennard-Jones potential. The Coulombic interactions were cut off at 1.0 nm and long-range electrostatic interactions were reincorporated using PME. The neighbor list in all simulations were updated every 10 simulation steps with a neighbor list cutoff of 1.1 nm. All MD and PT-WTE simulations used a 2 fs time step and LINCS [31] to constrain bonds to their equilibrium values. Center of mass translation is removed every 1000 simulation time steps.

The final frames from the heating simulations were used as the initial coordinates for the production simulations for each system. The D region simulations were run in the NPT ensemble with the Berendsen [32] barostat and a global stochastic thermostat [33] keeping the average pressure and temperature at 1 bar and 310 K, respectively. Replicas in the PT-WTE simulations also used this global stochastic thermostat. NPT simulations of the four D region systems were run for a total of 350 ns each and an additional two replicate simulations were run for each system for 200 ns after repeating the initial heating simulations with randomized initial velocities. These repeat simulations were used to verify the observations made in the longer production simulations. The PT-WTE simulations (more details follow) of the αC-subdomain were run for a total of 300 ns per replica for both the oxidized and non-oxidized systems. This resulted in aggregate simulation times of 8.4 and 3.0 µs for the αC-subdomain and D region, respectively.

### Enhanced sampling with PT-WTE

Detailed descriptions including underlying assumptions and the simulation protocol for well-tempered ensemble and PT-WTE are available elsewhere [34], [35]. Here we present a brief summary of this enhanced sampling method, our application of it, and distinguish the methodology applied in contrast to our recent publications [35], [36].

The metadynamics algorithm [37] is typically used as an enhanced sampling method for overcoming large energy barriers by biasing the collective variables (CVs) that govern important modes of motion and forcing the system to explore new areas of phase space with respect to these CVs. Collective variables are functions of the atomic positions of the system that can be calculated at any of the simulation's time steps. A simple example of a CV is the distance between two atoms and a more complex example is the system's potential energy (which is evaluated at every simulation time step). The bias implemented in metadynamics takes the form of small Gaussian-shaped hills, centered at the current value of the CV, and is added to the system's Hamiltonian every *τ* time steps. These hills gradually fill the local free-energy surface in the form of a history dependent bias. The well-tempered extension to metadynamics [38] limits the amount of bias that can deposited in any region of the CV-space by allowing the hill height to decay exponentially with the amount of bias deposited previously at the given CV value. Subsequently the bias potential for well-tempered metadynamics with a single collective variable (denoted *s*) takes the form of:
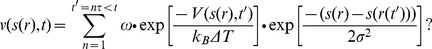
(1)


The initial hill height – in units of energy/mole – is denoted by *ω* and is modified by the exponential term containing the current value of the bias potential. The final exponential term within the summation describes the Gaussian hill of σ width that is added every *τ* time steps. Δ*T* represents a user-defined virtual temperature that in practice is implemented as the bias factor:
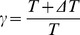
(2)


The PT method utilizes multiple non-interacting replicate simulations that are spaced over a distribution of temperatures. These replicas periodically exchange coordinates according to the Metropolis criterion [39] in a way that preserves detailed balance and canonical sampling at each temperature. The method is useful for enhanced sampling of slow degrees of freedom without prior distinction or specific bias of the important modes of motion. However, the PT method is burdened by high computation cost due to energy fluctuations scaling proportionally to N^1/2^, whereas the average potential energy of a system scales on the order of N. This leads to the requirement of large numbers of replicas to cover a small temperature range and maintain satisfactory exchange frequency in larger systems. It is well-known that combining PT and metadynamics (PTMetaD) can accelerate sampling and convergence [40], however this method does not address the severe limitation imposed by large system sizes.

**Figure 2:**
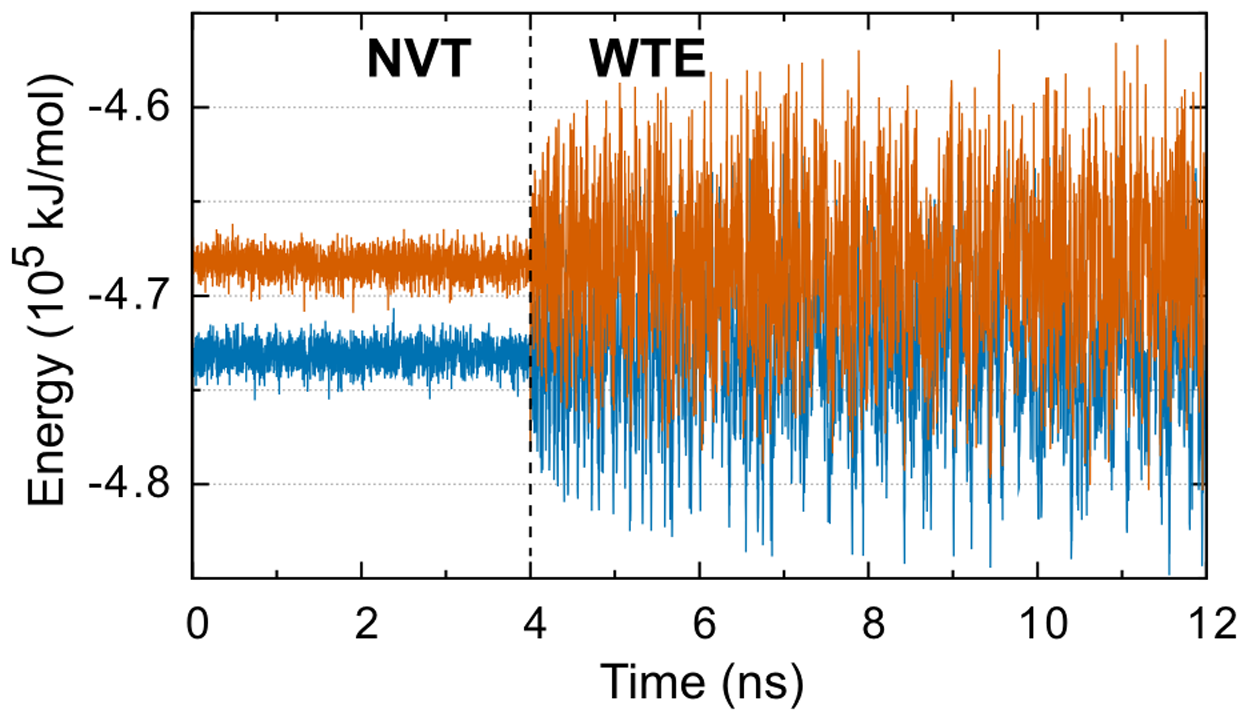
Energy fluctuations are increased by WTE bias: comparing the lowest two temperature replicas (310 K, blue and 319 K, orange) before (left) and after (right) the addition of WTE bias. Data was collected during the initial simulations used to determine the appropriate temperature spacing. The NVT and WTE data are taken from independent simulations, without and with the addition of PE bias, respectively, and do not represent a continuous simulation.

Recent exploration of the use of the systems potential energy as a biased CV with well-tempered metadynamics has shown that energy fluctuations in the system can be increased while preserving the canonical average [34] (illustrated in Figure 2). However, as systems under the influence of this metadynamics bias and augmented energy fluctuations do not conserve the canonical distribution, they have been dubbed to be in the well-tempered ensemble (WTE) and the scale of the fluctuations increases monotonically with the well-tempered metadynamics bias factor (γ). This advance has subsequently been applied toward amplifying the energy fluctuations during PT simulations and increasing the probability of replicas exchanging configurations. We have shown that PT-WTE can reduce the computational cost of PT simulations with negligible effects on canonically averaged observables for all-atom systems [35]. Another recent study by Sutto and Gervasio applied this method to explore the conformational free-energy surface of a receptor tyrosine kinase enzyme and examined the effects of single and double mutations [41]. In contrast to these other studies, here we present the first use of PT-WTE in an all-atom system without additional metadynamics bias on CVs related to our system configuration or modes of motion.

Prior to production PT-WTE simulations several parameters and system properties must first be determined. For this study we used a bias factor of 30, a temperature range of 310 to 450 K, a hill deposition rate of 200 simulation steps, an initial hill height of 4.0 kJ/mol, and a PT exchange attempt every 100 simulation steps. The σ value (Gaussian hill width) of 665 kJ/mol for the energy CV was determined from the standard deviation of the non-oxidized system's potential energy (PE) during a 2 ns NVT simulation at 310 K starting from the heated coordinates. The initial coordinates of the non-oxidized system are used to heuristically determine the two lowest temperatures for which PT-WTE simulation with a bias factor of 30 gives an average exchange probability of 30% after 10 ns (Figure 2). Determining the lowest two adjacent temperatures allows calculation of the number of replicas and correct spacing of replica temperatures to give constant exchange probability over the desired temperature range of 310 to 450 K [42], which was then used to space 14 replicas over this span.

PT-WTE simulations were implemented with the PT framework of GROMACS 4.5 along with the metadynamics plugin, PLUMED [43]. Each replica used initial coordinates corresponding to the final frame from the nearest temperature as generated during the initial system heating. In contrast to our previous publications, the current implementation of PT-WTE actively accumulated bias throughout the entire simulation and no preliminary simulation is used to create a static bias potential in the PE space. Due to the large system size and gap in PE between adjacent replicas there is initially no exchange between replicas during the early parts of the simulation (Figure 2). We stress that without the PE bias and the WTE methodology, neighboring replicas with this temperature spacing will not exchange configurations. The exchange probability converges to 30% within the first 5 ns and remains near this probability during the remainder of the simulation for all replicas.

Additional constraints were used to restrict the conformational explorations to structures with the individual hairpins intact. These restraints were implemented as two harmonic potentials on the Cα RMSD from the initial model for each hairpin individually. Therefore, the system is restricted to exploring configurations of the peptide with both hairpins intact yet not confined to the initial anti-parallel beta sheet structure. The harmonic potential was set to exert bias when the RMSD becomes higher than one standard deviation from the model structure (0.30 nm and 0.22 nm for the large and smaller hairpins, respectively), which was determined from the preliminary NVT simulations used to test the model stability, as described above. The spring constant for the potential was set to reach a value of 100 kJ/mol when the RMSD was two standard deviations from the initial model.

### Analysis

All analyses presented in this study were completed using the tools within GROMACS 4.5, the driver utility of PLUMED, and VMD 1.8 [44] in addition to custom scripts. Specifically, calculations involving the CV and PMF analysis used the driver utility of PLUMED. The functional form of the PMF, an estimate of the free-energy surface along a CV, is described elsewhere [45] and the CVs are described in the PLUMED documentation. Trajectory visualization and rendering of molecular representations was completed in VMD.

The clustering analysis used the g_rms and g_cluster tools from GROMACS. The g_rms tool was used to calculate an initial RMSD matrix from the lowest temperature trajectories using frames from every 20 ps after the first 10 ns of simulation, for a total of 14500 frames for each simulation. This matrix was used with g_cluster to cluster the frames by the Jarvis-Patrick method [46]. Jarvis-Patrick distinguishes clusters by comparing the intra-frame RMSD of frames within a cutoff and assigning cluster members if they share at least P frames within a cutoff of other cluster members. The cutoff was determined by examining the RMSD matrix to identify the typical distance between neighboring frames and was chosen to be 0.4 nm. Selection of such a large cutoff was necessary due to the flexibility of the protein when in its most extended conformations. The P value for the Jarvis-Patrick method, the number matching neighbors required to form a cluster, was chosen to be 10 by increasing the number from an initial 2 until the top 10 clusters represented clearly distinguishable conformations.

Bootstrapping was used to produce a confidence interval[47] for each bin in the PMF calculation. Specifically, we used every tenth frame (10%) from the full data set to form a reduced data set that was subsequently randomly sampled with replacement to recalculate the PMF estimates. This resampling and recalculation procedure was repeated until the standard deviation of each of the bin estimates converged within 10^−3^ kJ/mol. The bootstrap confidence intervals are displayed in the PMF plots as shaded regions surrounding the PMF calculated from the reduced data set without random sampling. We used a reduced data set to avoid oversampling and also repeated the bootstrapping procedure, further reducing the data set to every hundredth frame (1%), without any major changes in our results.

## Results

### D region

The fibrin D region has been shown to possess a feature critical in the ability of fibrin to aggregate and form proto-fibrils – hole ‘a’. The D region has also been shown to contribute in the lateral aggregation of the fibrils, but may not be critical to this process [17]. Its role in this process is hypothesized to involve hole ‘b’, an indented binding site located on the globular portion of the β chain of Fibrin, interacting with knob ‘B’, the N-terminal portion of the fibrin β chain after thrombin cleavage of the B-peptide. Figure 1A shows a representative snapshot of the modeled protein, with bound GHRP peptides and with methionine sulfoxide, and exemplifies the key location of βMet367 in relation to the ‘B:b’ knob-hole interaction. We have hypothesized multiple mechanisms by which methionine oxidized to methionine sulfoxide may interrupt the proposed mechanism that are amenable to investigation by MD simulation: methionine oxidation disrupts the conformation of the globular portion of the D region, disrupts the conformation of hole ‘b’, and/or creates an unfavorable interaction with knob ‘B’ and destabilizes its bound state. To test these hypotheses we performed 12 simulations of four different systems: the D region either with or without methionine oxidized to methionine sulfoxide and with or without bound knob ‘B’ surrogates. Analysis of the effects of oxidation on the protein structure and characteristic fluctuations were tracked by the Cα root-mean-square deviation (RMSD) and fluctuation (RMSF) over the course of the 350 ns NPT simulations in addition to the 200 ns replicate simulations.

**Figure 3:**
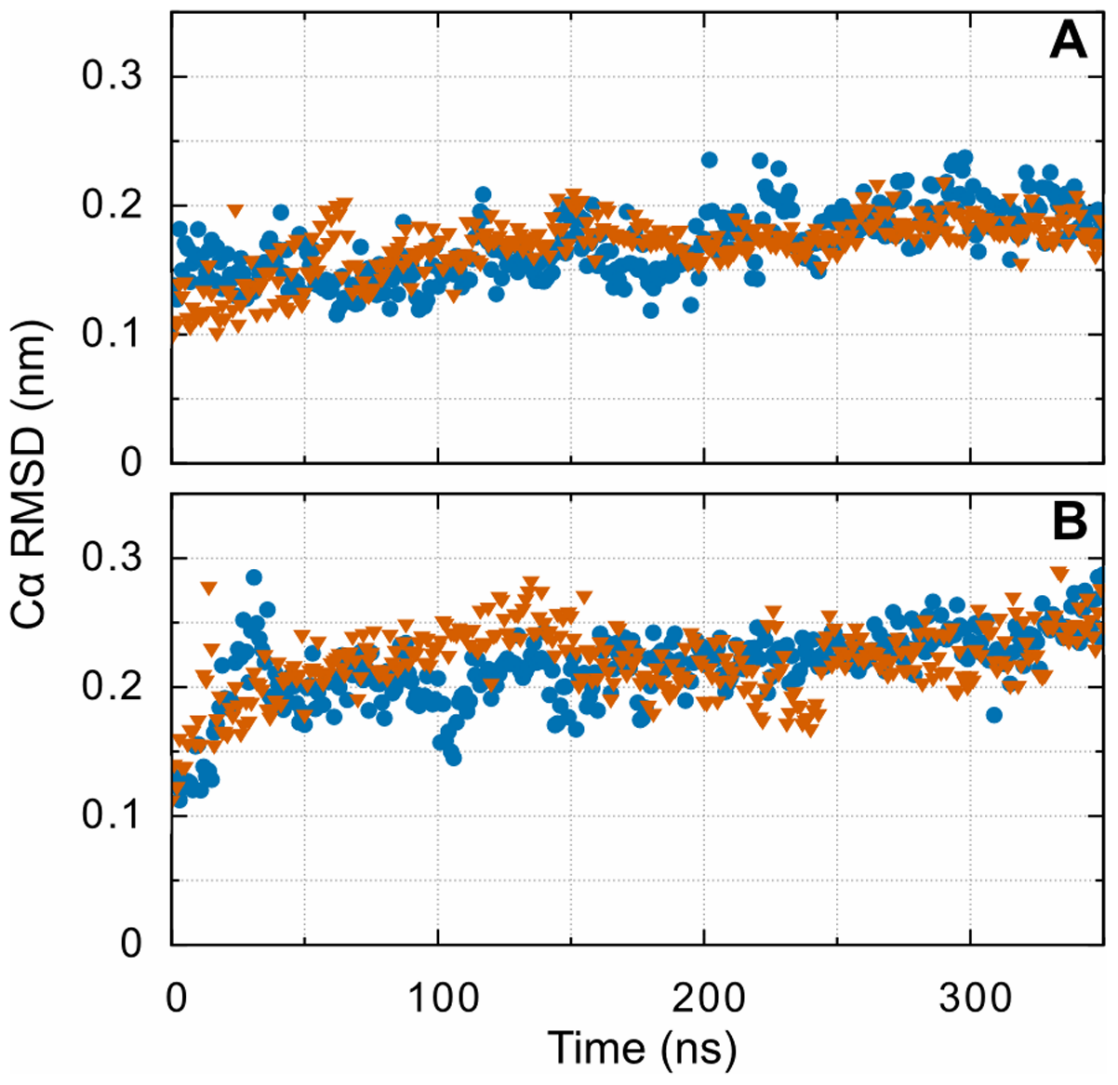
D region deviations from the initial structure highlighting the difference between oxidized (orange) and non-oxidized simulations (blue) for (A) the systems with bound GHRP peptides and (B) without bound GHRP peptides. The D region simulations show no augmented deviation created by βMet367 oxidation for either group. These features are confirmed in the replicate simulations, although these simulations show some variability in the final RMSD value.

Measuring the RMSD throughout an MD trajectory allows the overall deviation from the initial structure of the protein to be tracked. Comparing the Cα RMSD, calculated for and aligned to just the D region (residues β202-459 and γ143-393), between the systems with or without oxidation (Figure 3) shows that, after sharp increase typical in MD simulation of proteins, the D region equilibrates to a similar deviation from the initial structure regardless of the state of oxidation of βMet367. The final RMSD for the systems without the bound knob ‘B’ surrogates are also independent of oxidations albeit larger than the systems with the peptides docked. Although the final RMSD values change to a small degree, the replicate simulations verify that indeed the overall structure of the globular portion of the D region are similar between the oxidized and non-oxidized systems on the 350 ns time scale and that relatively few changes from the x-ray structure in the D region are observed.

**Figure 4:**
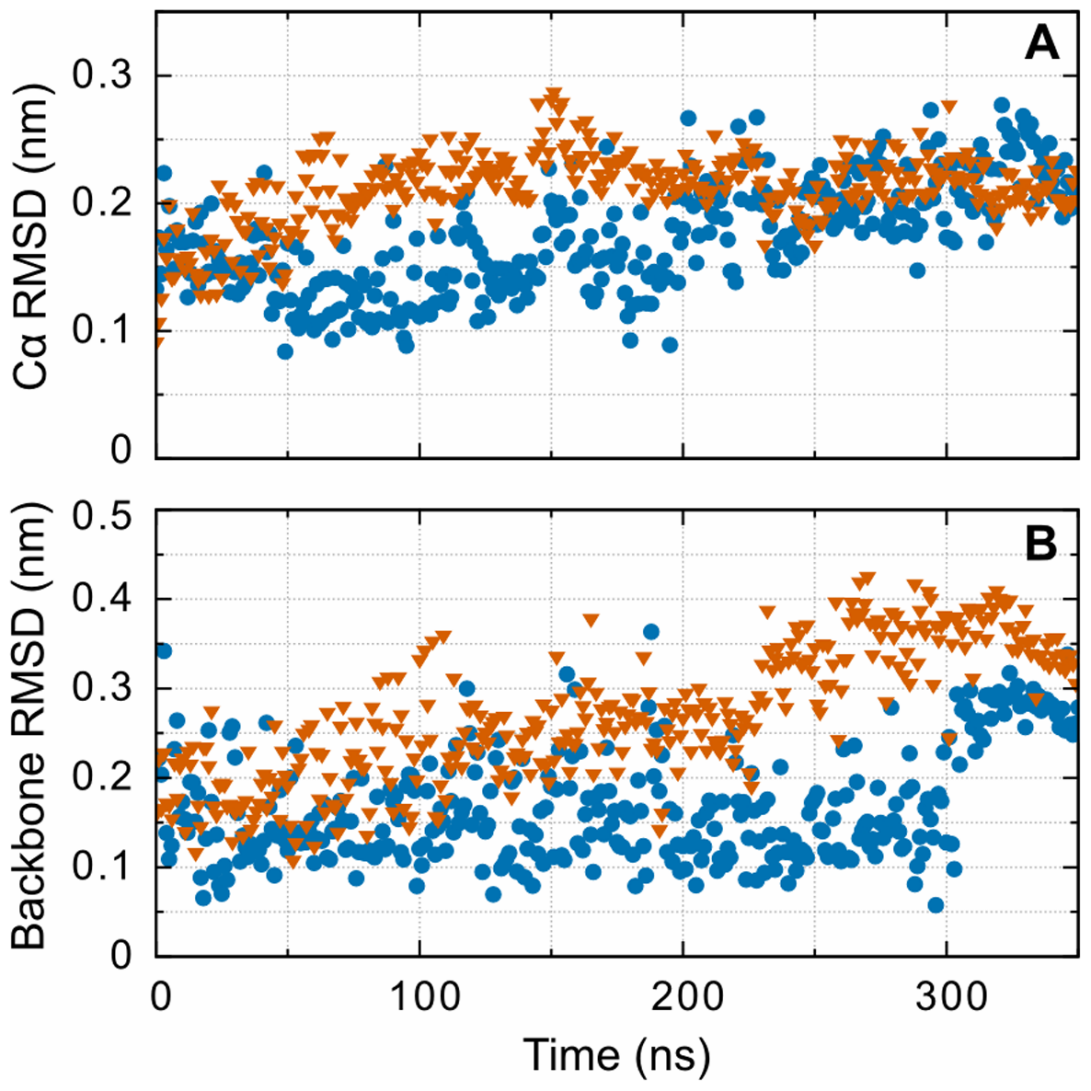
RMSD from the initial structure highlighting the difference between oxidized (orange) and non-oxidized simulations (blue) for (A) hole ‘b’ corresponding to residues β352-370 and β381-447 and (B) the GHRPL fragment bound to hole ‘b’. Note the difference in the vertical axis scale. Like Figure 3, the final RMSD after oxidation is similar to the non-oxidized systems. The sharp increase in backbone RMSD for the GHRPL peptide (knob ‘B’ surrogate peptide) is caused by the solvated proline and leucine backbones reorienting toward increased interaction with hole ‘b’.

In addition to the structural changes observed for the entire D region we used the trajectory after aligning to the D region to measure the RMSD of just hole ‘b’ and monitor the structural deviations related to hypothesis surrounding altered confirmations of hole ‘b’. This portion of the experimental structure [18] corresponds to residues β352-370 and β381-447. These residues were determined by their proximity to the knob ‘B’ peptide in the experimental structure. Similar to the results for the whole D region, hole ‘b’ exhibits a similar final value for both the oxidized and non-oxidized systems (Figure 4A). Additionally, the RMSD of these regions without bound GHRP peptides show a larger deviation from the initial structure that is also independent of oxidation. This feature is also seen when performing a similar calculation for hole ‘a’ (residues 289-307and 320-379 γ). These observations, when combined with the RMSF analysis described below, indicate that the increased RMSD in the whole globular domain can be at least partially ascribed to the increased fluctuations of holes ‘b’ and ‘a’ in the absence of a bound knob ‘B’ surrogates.

The backbone RMSD of the knob ‘B’ fragment itself is used to measure displacement and binding rearrangement of the GHRPL peptide bound to the fibrin hole ‘b’ when calculated from the trajectory aligned to the D region. Backbone RMSD is used here to increase the number of atoms used when calculating the displacement, reducing the noise observed in the Cα RMSD. Examining the results for this calculation (Figure 4B) reveals that knob ‘B’ peptide is remarkably stable when bound to hole ‘b’ of fibrin, with only a slight increase in final RMSD in the oxidized system. Repeating this calculation for the knob ‘B’ peptide bound to hole ‘a’, a location physically removed from the oxidized βMet367, along with the results from the replicate simulations reveal that this increase is negligible and likely unrelated to methionine oxidation. However, analysis of the trajectory does reveal differences in how βMet367 interacts with surrounding residues upon oxidation. Viewing the trajectory reveals that, in the non-oxidized system, βMet367 interactsand stays in close contact with the histidine and proline of the GHRP peptide. In the oxidized system, this changes from a primarily hydrophobic interaction to hydrogen-bonding-like electrostatic interaction between the methionine sulfoxide oxygen and either the backbone nitrogen between histidine and arginine during the first half of the simulation or to the histidine side chain later in the simulation. The sharp, nearly 0.1 nm increase in RMSD for both of these systems, near the end of the simulations, is due to the backbone of residues proline and leucine, of the knob ‘B’ peptide, reorienting toward increased interaction with hole ‘b’, as opposed to being primarily solvent exposed. In the oxidized system this leads to hydrogen bonding between the knob ‘B’ C-terminal carboxylate and the leucine side chain with βLys392 and βLeu386, respectively. In the non-oxidized system this leads to closer contact between the βMet367 side chain and the histidine and proline of knob ‘B’.

**Figure 5:**
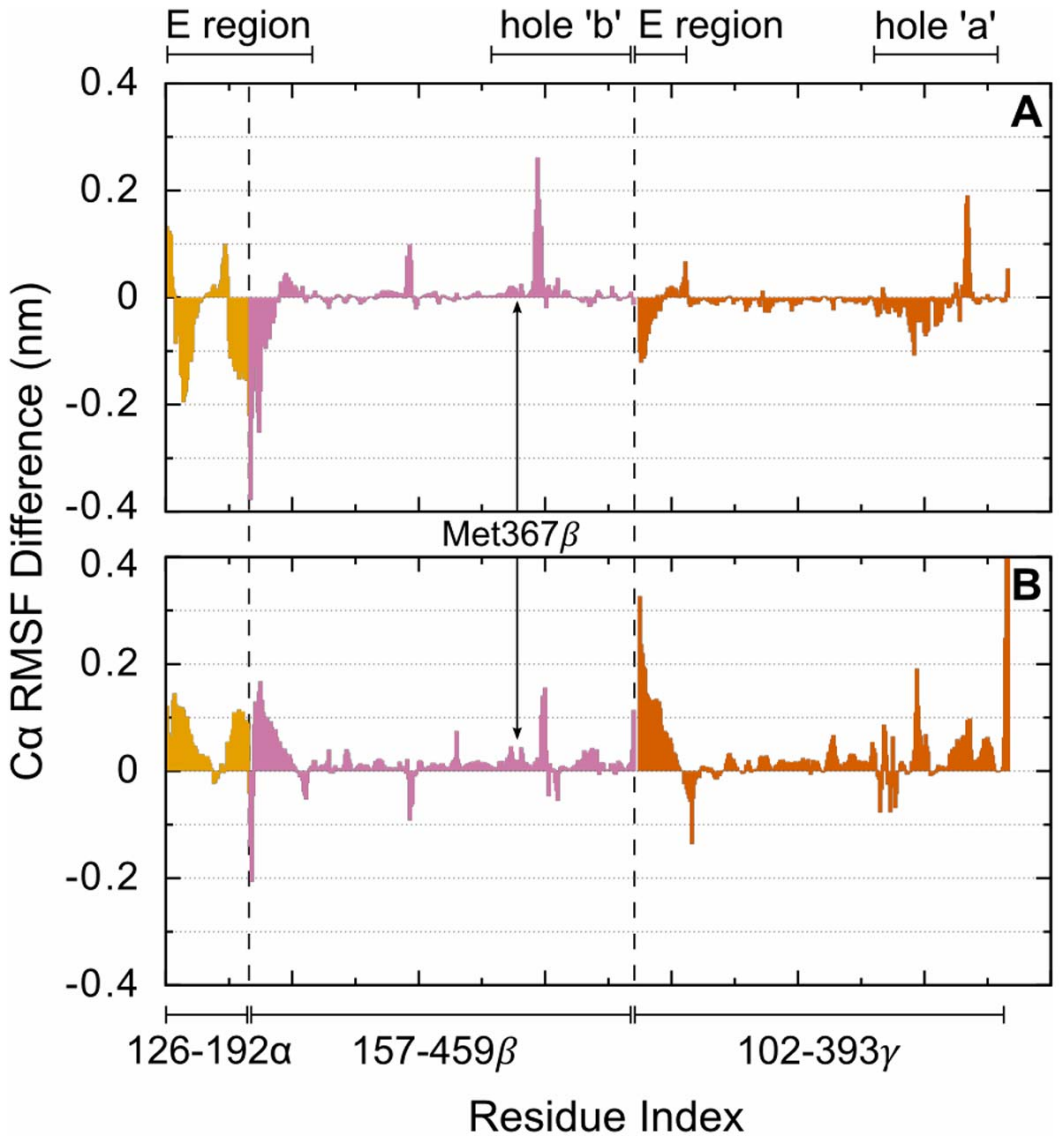
Cα fluctuation differences between the oxidized and non-oxidized simulations for systems (A) with bound GHRP peptides and (B) without bound GHRP peptides. Colors differentiate the multiple protein chains that comprise the modeled system and correspond to the colors used in figure 1A. Dashed lines indicate the division between each chain. Positive and negative values indicate increased fluctuations in the oxidized and non-oxidized systems, respectively.

Like the structural deviations described by RMSD, the characteristic fluctuations of the protein can be described by the Cα RMSF calculated over the equilibrated portion of the trajectories. Simulations were deemed equilibrated after the relatively sharp increase in RMSD observed during the first several nanoseconds. Taking the equilibration period to be the first 10 ns of NPT simulation ensured that each measurement contained the same number of structures. To aid in the description of changes in RMSF between the systems, per-residue difference in the RMSF, rather than the magnitude of the individual RMSF calculations, was plotted by subtracting the results of the non-oxidized from the oxidized systems (Figure 5). Therefore, positive values indicate larger fluctuations in the oxidized system while negative values indicate larger fluctuations in the non-oxidized system.

Figure 5 highlights several regions of the enzyme with increased fluctuations or large differences in fluctuations upon oxidation. The coiled-coil E region of the protein shows the largest RMSD and RMSF amongst all the simulations particularly in residues near the point of truncation in the crystal structure. This section of the peptide consists of 4 helical coils, which are contributed from each of the α, β, and γ chains, and is stabilized by multiple, inter-chain disulfide bonds. However, these disulfide bonds do not prevent translation of the helix bundle relative to the D region nor create stability near the point of truncation. These domain motions in the E region lead to relatively large deviations in the protein structure that are likely stochastic and unrelated to methionine oxidation. The other remarkable regions, in terms of structural fluctuations, are holes ‘a’ and ‘b’ whether with or without bound knob ‘B’ surrogate peptides. In all simulations these residues exhibit largest fluctuations of all the D region and exhibit some structural reorganization from the crystal structure toward solvating several hydrophilic amino acids. This is particularly evident in the 0.24 nm increase in RMSF at aspartic acid β383 for the oxidized system with bound knob ‘B’ over its non-oxidize counterpart. There is a similar increased RMSF and increased RMSF difference for this residue in the system without knob ‘B’ peptides but the difference seems comparatively insignificant. Viewing the trajectory of the oxidized system with bound GHRPL shows that this aspartic acid residue moves from being partially buried near the surface of the protein in a network of side-chain-to-backbone hydrogen-bonding interactions toward becoming fully solvent exposed and forming multiple short-lived interactions with nearby hydrophilic amino acids. This aspartic acid and its sequence neighbors form interactions with the knob ‘B’ peptide toward the end of this simulation but are beyond the non-bonded cutoffs from βMet367. Overall, comparing the Cα fluctuations reveals little to no evidence that oxidation increases the fluctuations of any region of the peptide at the 340 ns time scale.

### αC-subdomain

The αC-subdomain displays a dynamic character that is thought to be critical toward its role in lateral aggregation of fibrin [48], [49]. The dynamic character has been hypothesized to include an “opening” of the double hairpin structure, characterized by a loss of contacts between the two hairpins and a hinge-like transition toward a linear and extended state. This feature is hypothesized to allow formation of large beta sheet structures with other fibrin molecules [48]. Opening and closing of the hairpins is explored in this study through the enhanced sampling added by the PT-WTE method. Due to the nature of the large structural variations upon opening and the frequency of exchanges with neighboring replicas, the trajectory for a single temperature contains a large and discontinuous variety of structures. When combined with the increased number of accessible configurations after the hairpins lose contact, this feature makes analysis by RMSD and RMSF less effective in describing the effects created by methionine oxidation. Therefore, in contrast to the analysis presented for the D region, the PT-WTE simulations were analyzed using RMSD clustering and supported by the potential of mean force (PMF) of multiple CVs. These analyses allow a description of the most preferred structures of the αC-subdomain and require the enhanced sampling of peptide conformations provided by PT.

**Figure 6:**
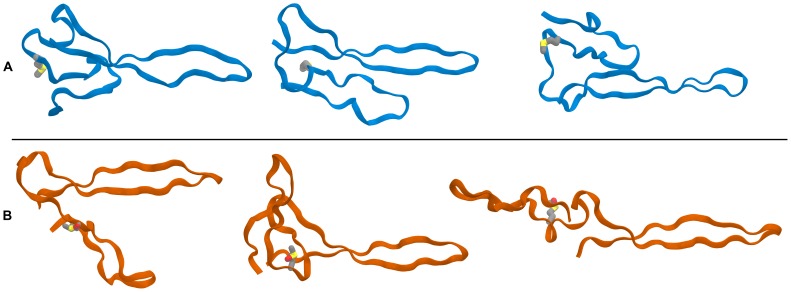
Ribbon representations of the cluster center from top three most populated clusters of the (A, blue) non-oxidized and (B, orange) oxidized αC-subdomain simulations. Clustering was calculated from the inter-frame Cα RMSD of the 310 K replicas. The ranking of the structures decreases from left to right; the leftmost structure is most populated cluster for each system. The side-chain of αMet476 is shown in each representation. The flexibility of the “hairpin-connecting region” affords a large variety of associations between the two hairpins and permits a hinge-like transition toward an extended “open” conformation (similar to the third ranking cluster of the oxidized system).

Jarvis-Patrick clustering analysis was applied to the backbone RMSD between frames after aligning to the larger of the two hairpins, corresponding to residues 443 to 465 α. This alignment creates increased RMSDs for protein structures at different degrees of extendedness and can still distinguish structures where the hairpins are still in contact (closed) yet in a different configuration compared to the original model. The heterogeneity of observed structures in the fully extended (open) conformation required a high clustering cutoff. Clusters represent the most likely conformations of the peptide according to the conformations explored at a given temperature. Indeed the top ten most populated clusters for the oxidized and non-oxidized systems contain 44% and 48% of the frames used in the clustering calculation, respectively. Viewing the structures included in each cluster along with the cluster representative, which correspond to the “center-most” member of the cluster, reveals that of the top ten most populated structures of each simulation show a discrepancy in preference for the open versus closed conformation between the two simulations. The most populated cluster for both the oxidized and non-oxidized systems at 310 K are similar in configuration to the original model. Figure 6 shows that the first and third highest populated clusters in the oxidized system correspond to a partially open and fully extended configuration, respectively. In contrast, the non-oxidized system the top 4 most populated clusters indicate multiple closed states are most prevalent in the trajectory. The fifth most populous cluster, representative of 7% of the clustered frames, exhibits the first open structure in the non-oxidized system. Of the top ten clusters in each system, the oxidized system has 4 clusters representing open or partially open structures (23% of the clustered frames) while the non-oxidized system has 2 (11% of the clustered frames). These data indicate that either methionine oxidation disturbs the stability of the closed configurations or stabilizes particular opened structures allowing them to contribute clusters with relatively higher population.

**Figure 7:**
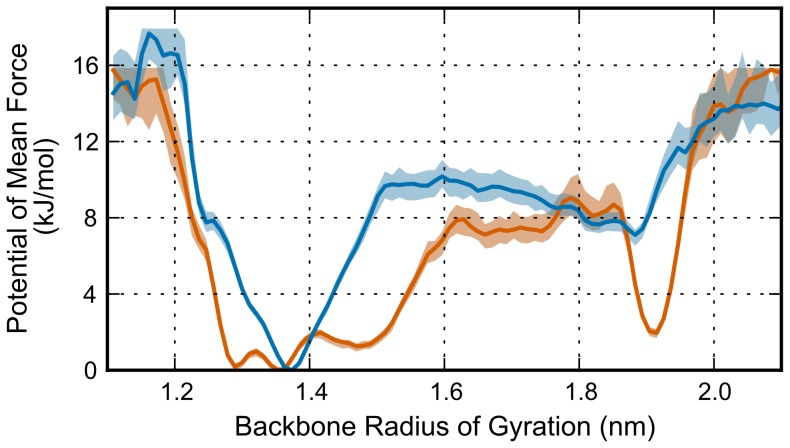
Comparing the PMF of the backbone radius of gyration of the entire αC-subdomain between the oxidized (orange) and non-oxidized (blue) systems. PMFs were calculated from trajectories of the 310 K replicas using every tenth frame to avoid oversampling. The well near 1.9 nm radius of gyration corresponds to fully extended structures similar to the third ranking cluster of the oxidized system, which can be seen in figure 5. Shaded regions represent a 99% confidence interval calculated using bootstrapping.

Several CVs describing aspects of the protein structure were calculated over the trajectories at each temperature. The potential of mean force (PMF) were in turn calculated from the histogram of CV values. These estimates of the free-energy surface along the CV coordinates entail characteristics of the preferred configurations for the αC-subdomain over the 300 ns PT-WTE simulations and are rescaled to set the lowest energy feature to zero. PMF calculations were recalculated at 80–100% of the trajectory in increments of 5% to check for convergence of the estimated free-energy surface features. We used the backbone radius of gyration of the peptide to examine the stability toward the hypothesized native structure, which has a low radius of gyration. Figure 7 compares the calculated PMF at 310 K between the oxidized and non-oxidized peptide. The PMF for the non-oxidized system describes a deeper well near a radius of gyration typical in closed configurations when compared to the oxidized system. The corresponding well for oxidized system is much broader in the radius of gyration, showing that a variety of structures – both slightly lower and higher in radius of gyration – contributes to the closed configuration in the oxidized system. Furthermore, there is a distinct well in the PMF for the oxidized system at large radius of gyration. This well is not as distinguished in the non-oxidized system. The frames corresponding to this value of radius of gyration are similar to the fully extended configuration seen in the third most populous cluster from the oxidized system, as shown in figure 6. These features of the free-energy surface along the radius of gyration indicate that methionine oxidation has caused a relative destabilization of the most preferred radius of gyration in the oxidized system and exhibits a higher proportion of open structures, particularly those similar to the third most populous cluster from the oxidized system. When combined with the comparison between the clusters calculated for each system, this analysis reinforces the observation that oxidation of methionine contributes to a more open configuration or possibly destabilizes the structure corresponding to the original model.

Viewing the trajectory and the cluster centers shows that for both systems the hairpin-hairpin interaction seen in the closed structures is not restricted to the anti-parallel beta sheet like conformation seen in the original model. The structures in the closed conformation reveal that a variety of hairpin-hairpin interactions occur with multiple orientations of the hairpins, including “face-to-face” hairpin stacking and side-by-side beta sheet on the opposite side compared to the starting model. The multiple orientations allowed by the hairpins are supported through the high proportion of polar or charged amino acids comprising the hairpin regions of this peptide. Of the 56 amino acids comprising the modeled system, 31 have side chains capable of forming hydrogen bonds. These amino acids associate through hydrogen bonds with other side-chains, forming salt-bridges, and with the backbone oxygen and nitrogen atoms. This aspect of the composition of this subdomain allows for stabilizing interactions from multiple orientations of the two hairpins beyond the backbone hydrogen-bonding characteristic in beta-sheets.

**Figure 8:**
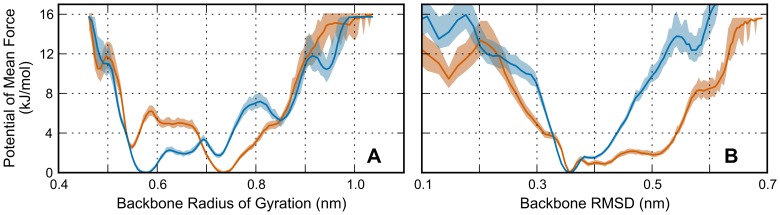
Comparing the PMF of the backbone (A) radius of gyration and (B) RMSD of the hairpin-linking region between the oxidized (orange) and non-oxidized (blue) systems. PMFs were calculated from trajectories of the 310 K replicas using every tenth frame to avoid oversampling. Both of these plots exhibit features indicative of a more extended linking region in the oxidized system. Shaded regions represent a 99% confidence interval calculated using bootstrapping.

The sequence of amino acids forming the link between the hairpins, which includes αMet476, confers much of the flexibility allowing the wide variety of structures seen in the closed state and serves as the hinge in the opening transition. Visual comparison between the cluster centers in both simulations shows that different orientations, especially when comparing between open and closed structures, are supported by extension and reorientation of the structure in this hairpin-linking region. We calculated the PMFs for the backbone RMSD, calculated from the initial model, and the backbone radius of gyration CVs for the hairpin-linking region. Figure 8 shows that, when comparing the estimate of the free-energy surface for these collective variables between the two systems, there are differences created by methionine oxidation. Specifically, the RMSD of the linking region in the oxidized system readily accesses a much broader distribution of structures compared to the linking region. This is interpreted from the PMF by a lower energy, relative to the minimum, up to 3.2 nm radius of gyration when compared to the PMF of the non-oxidized system. This feature of the PMF indicates that a larger variety of structures, particularly those that deviate more from the initial model, are relatively more stable in the oxidized system. The PMF calculated from the backbone radius of gyration of the linking region supports this observation and reveals that the most visited configurations during the oxidized simulation have over a 0.15 nm increase in radius of gyration over the most visited conformations of the non-oxidized system. This amount may appear small at first glance but given the relatively small size of the connecting region, a 0.15 nm corresponds to significantly different structures. In summary, the features of the PMFs for these two CVs indicate that the oxidation of methionine alters the conformations of the connecting region toward increased deviation from the original model and an increased extendedness.

## Discussion

On the 350 ns time scale, the MD simulations of the fibrin D region provide no indication of an alteration in the D region structure or characteristic fluctuations – either as a whole or when confining our analysis to hole ‘b’. Over the same time scale these simulations do not support the hypothesis that methionine oxidation disrupts the stability of the bound knob ‘B’ peptide. However, the enhanced sampling simulations indicate clear differences in the preferred configurations of the αC-subdomain indicative of oxidation influencing the structure's preference away from the assumed native conformation. We ascribe the disruption of the αC-subdomain to observed changes in the conformation of the hairpin-linking region, the segment of the subdomain that contains αMet476.

Experimental investigations detailed in the current literature have established that the αC-domain plays a critical role in the lateral aggregation of fibrin [14], [50]. Additionally, the experiments conducted by Weigandt et al. [13] show that oxidized fibrin gels exhibit similar properties to fibrin gels without functional αC-domains [14]. This work contributes to a growing body of evidence that the oxidation of the αC-domain is one likely contributor to the mechanism of disrupted lateral aggregation of oxidized fibrin. However, the molecular mechanism of αC-domain influenced aggregation has yet to be established. One hypothesis is that the double hairpin structure of the N-terminal subdomain forms extended beta-sheet structures through its interaction with the same subdomain of nearby fibrin molecules [48]. The disruption of the preferred conformation of this subdomain could, thus, influence its ability to interact favorably in this interaction. However, until the mechanism of αC-domain influenced lateral aggregation is known, the importance of the native structure in this mechanism cannot be established.

Reactive oxygen species (ROS) and ROS-generating oxidases are known to play important roles as signaling molecules in the vasculature. Many cellular components including platelets, endothelial cells, and polymophonuclear cells release ROS in response to tissue injury and ischemia. Lipoxygenases and cyclooxygenases in platelets can make superoxide via NADPH oxidases, nitric oxide from nitric oxide synthases, and ultimately peroxynitrite via interaction of superoxide with nitric oxide [15]. Specific to our study, polymorphonuclear cells (neutrophils) produce hypochlorite by the interaction of myeloperoxide and hydrogen peroxide, which is also key host-defense mechanism mediating bacterial killing by the formation of methionine sulfoxide in bacterial membrane proteins [51]. Therefore, our results identifying a change in fibrin αC-domain structure as a result of selective methionine sulfoxide formation perhaps suggest that neutrophil-specific oxidation can also regulate fibrin clot structure. Further evidence for this process taking place in vivo was found by Heffron et al, after inducing experimental endotoxemia in healthy humans. The authors found that endotoxemia-induced fibrinogen nitration was preceded by a stable increase in myeloperoxidase concentration in plasma [52]. Interestingly, neutrophil-mediated methionine oxidation by hypochlorite can also dose-dependently inhibit other important coagulation proteins including activated protein C [53], and thrombomodulin. In the case of thrombomodulin, inhibition has been mapped to oxidation of a single methionine residue (Met388) [54]. Taken together, the influence of methionine sulfoxide formation on the key regulators of protein C, thrombomodulin, and fibrinogen also suggests a possible wider role for oxidative stress as a mechanism of regulation of coagulation function by neutrophil inflammation.

Several assumptions were required to conduct targeted simulation studies of oxidation's effect on fibrin. In particular, our investigation into the αC-subdomain hypotheses requires the assumption that the human homology model of the bovine NMR structure is an appropriate representation of this subdomain. Furthermore, we have restricted our conformational exploration of this peptide by restraining the secondary structure of each hairpin near its initial configuration. This was done to avoid exploration of the complete conformational ensemble of this peptide – an endeavor that would require unreasonably long simulation times. Therefore, our simulations cannot investigate an alternative hypothesis: that the oxidation of αMet476 completely disrupts the preferred fold of the αC-subdomain through its interactions with nearby residues. Additionally, the D region simulations are not sufficient to investigate another alternative hypothesis: that oxidation of βMet367 interferes with knob ‘B’s initial docking with hole ‘b’. Another consideration is that oxidation within the D region occurs in an area of high stability with well-defined secondary structure, whereas the oxidation in the αC subdomain is in a region comprised of a flexible loop, which is expected to have greater mobility.

Our results and untested hypotheses suggest several opportunities for future simulation work to further investigate methionine oxidation of fibrin. In particular, the hypothesized aggregation mechanism involving multiple αC-domains forming extended structures suggest that methionine oxidation might interrupt lateral aggregation by disruption of this interaction. Additionally, the altered conformational preference described in this study after methionine oxidation could influence the interaction between two αC-subdomains. Docking studies or metadynamics simulations could be used to test this hypothesis by studying the interaction of pairs of αC N-terminal subdomains with and without methionine oxidation. Furthermore, the ‘B:b’ knob-hole interaction could be further examined by a metadynamics docking study to calculate the free energy surface of a knob ‘B’ surrogate docking to an oxidized hole ‘b’. Additionally, future computational work using GPUs or millisecond-time MD simulation machines [55] could contribute in both of these areas.

It may also be useful to seek to identify similar oxidative modifications in diseases where oxidative stress and coagulopathy are known to co-exist. Hemorrhagic shock from blood loss in trauma is a prime example of such a condition. Tissue hypoxia from blood loss paradoxically increases oxidation both from mitochondria [56] and from activation of NADPH oxidases [57]. Coagulopathy is also almost immediately present in almost 25% of severely injured trauma patients, requires a high degree of cardiovascular shock with tissue hypoperfusion, and results in a 4–6 fold increased mortality [58], [59], [60], [61], [62]. Moreover, functional fibrinogen concentration and fibrinolysis are both strongly associated with mortality in trauma patients [63], [64] and both can be strongly influenced by methionine oxidation. However, further study is required to delineate any potential role for fibrinogen oxidation in the pathophysiology of traumatic coagulopathy.
